# Color‐Contrast Adjustment With a 3D Head‐Up System to Aid Capsulorhexis After ICG Staining: Single‐Patient Case Report From a Tertiary Center

**DOI:** 10.1155/crop/5414207

**Published:** 2026-04-25

**Authors:** Yukihisa Takada, Takayoshi Sumioka, Nobuyuki Ishikawa, Shizuya Saika

**Affiliations:** ^1^ Department of Ophthalmology, Wakayama Medical University School of Medicine, Wakayama, Japan, wakayama-med.ac.jp

**Keywords:** 3D head-up surgery, anterior capsular staining, capsulotomy, cataract surgery, indocyanine green

## Abstract

We report a single case in which continuous curvilinear capsulorhexis (CCC) was safely completed using NGENUITY in 3D head‐up surgery (HUS) during cataract surgery. Visibility of the dye‐stained anterior capsule was improved during the CCC procedure. An 84‐year‐old female underwent a planned extracapsular extraction on December 2022, in HUS using NGENUITY for a Grade 4 cataract classified by the Emery–Little classification. After staining the anterior capsule with 0.5% indocyanine green (ICG) for 5 s, the anterior chamber was washed to reduce the risk of endothelial damage. Then, CCC was performed by adding red enhancement to a monochrome‐filtered image using the color contrast adjustment function of NGENUITY. The contrast of the anterior capsulotomy margin appeared sharper than in the normal setting, which enhanced the visibility of the capsulotomy margin of the anterior lens capsule. This allowed CCC to be completed smoothly and safely. Subsequently, the surgery was performed as usual and the intraocular lens (IOL) was fixed in the capsular bag to complete the procedure. One month after surgery, the fixation and position of the IOL were stable. Color channel adjustment in a 3D head‐up system improved the visibility of CCC margins after ICG staining in advanced nuclear cataracts. This technique may be useful in cases with a poor red reflex. Future prospective studies using objective contrast evaluation are needed.

## 1. Introduction

In cases of cataracts with advanced nuclear sclerosis, the lens nucleus is sometimes dense and bulky. This could disturb performing a larger continuous curvilinear capsulorhexis (CCC) that is beneficial to safely deliver the nucleus during extracapsular cataract extraction (ECCE). The relative difficulty of transillumination through the operating microscope disturbs the recognition of the anterior capsule margin during CCC. Anterior capsule staining is effective for ensuring visibility in cases with poor transillumination caused by advanced nuclear sclerosis, mature cataract, or corneal opacity using reagents such as indocyanine green (ICG) [1], trypan blue (TB) [2], and brilliant blue G (BBG) [3].

In recent years, 3D head‐up surgery (HUS) has developed remarkably in ophthalmic surgery, mainly in the field of vitreous surgery. In this system, a stereoscopic digital camera projects the surgical field onto a large 3D monitor, allowing the surgeon to adjust the color and contrast in the digital images. It has been reported that internal limiting membrane peeling, performed using the abovementioned staining reagents, can improve visibility better than before by adjusting the color contrast with image processing [4]. Although the effectiveness of HUS in cataract surgery has also been reported [5], there are no reports on the effectiveness of HUS for CCC creation after staining the anterior capsule. Several previous studies have attempted to improve anterior capsule visibility using various approaches, such as enhanced coaxial illumination, endoillumination, and retroillumination techniques to improve contrast between the capsule and nucleus, and the use of digital visualization systems in dense cataracts [5, 6]. Although these conventional methods can improve visualization to some extent but may be limited in cases with dense nuclear sclerosis or weak red reflex, color contrast adjustment by digital imaging may offer further benefits. However, none of these studies have focused on color contrast adjustment after anterior capsule staining.

We report a single case in which we performed image processing in HUS using NGENUITY (Alcon, Fort Worth, Texas, United States) for cataracts with advanced dense and bulky nucleus, allowing us to more safely complete CCC by further improving the visibility of the anterior capsule margin after dye‐staining the capsule.

## 2. Case Presentation Patient

### 2.1. Patient

An 84‐year‐old female with Lewy body–type dementia was referred to the Ophthalmology Department of Wakayama Medical University Hospital (hereinafter, referred to as our department) on September 2022, for the purpose of bilateral cataract surgery.

### 2.2. Findings at the Initial Examination in our Department Clinic

There were no abnormalities in the anterior segment of either eye. Her visual acuity was 0.01 (0.05 × S − 8.00D: C − 5.00DA × 120^°^) in her right eye and 0.01 (0.15 × S − 7.00D: C − 5.00DA × 80^°^) in her left eye. The cataracts were at Grade 4 on the right and Grade 3 on the left according to the Emery–Little classification (Figure 1a,b), disturbing retroillumination. The fundus of her right eye was visually unclear. There were no abnormalities in the fundus of her left eye.

Figure 1Anterior segment photos (a, b) Preoperative photos. (c, d) Postoperative photos.

**Figure:**
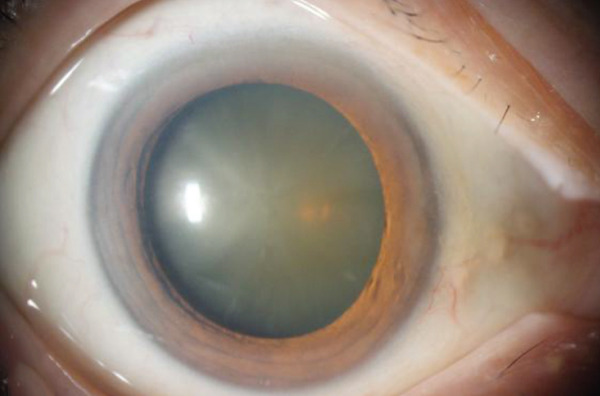
(a)

**Figure:**
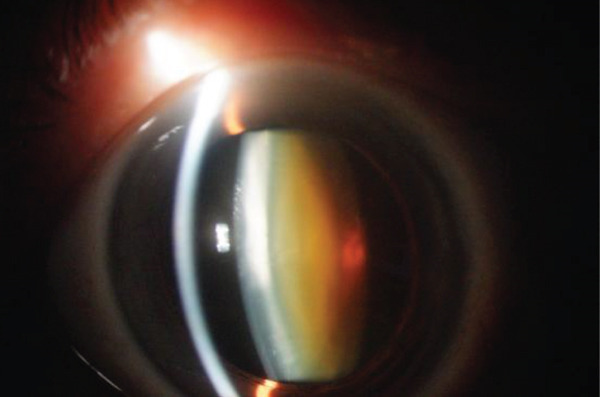
(b)

**Figure:**
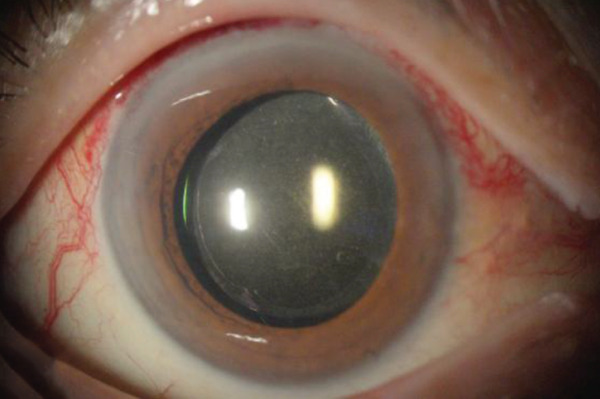
(c)

**Figure:**
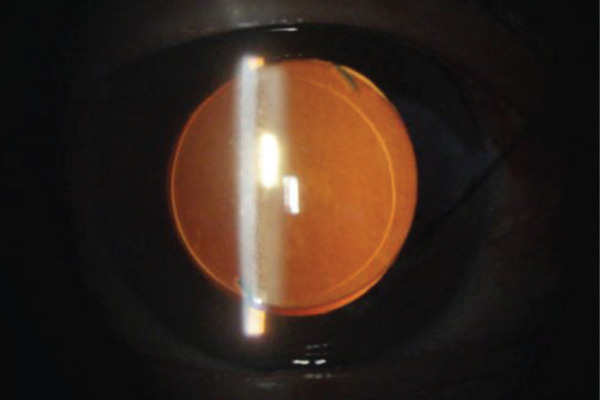
(d)

### 2.3. Clinical Course

A planned ECCE was performed on her right eye at our department on December 2022. We selected the surgical method taking into consideration the possibility of the dense and bulky nucleus and age‐related fragility of the zonules. In this case, we anticipated that poor transillumination and a weak red reflex due to the dense, Grade 4 nucleus would make visibility of the anterior capsule margin particularly difficult. Therefore, we performed 3D HUS using the cataract surgery device CENTURION (Alcon, Fort Worth, Texas, United States), by connecting NGENUITY to the operating microscope OPMI Lumera700 (Carl Zeiss AG, Oberkochen, Germany).

The surgery was performed by creating a side port at 10 o′clock and staining the anterior capsule with 0.5% ICG diluted with BSS Plus (Alcon, Fort Worth, Texas, United States) (for 5 s), and the dye was immediately washed with 3 mL of BSS Plus in order to reduce the risk of endothelial damage. The anterior chamber was then filled with a low molecular–weight viscoelastic substance, a purified sodium hyaluronate preparation (OPEGAN 0.6 eye viscoelastic agent 1%, Santen Co. Ltd., Tokyo, Japan). In order to improve the visibility of the anterior capsulotomy margin during CCC, the NGENUITY image was changed from a normal image to a monochrome filter image with red tone emphasized (details are described below). CCC was then performed using a 26G cystotome. After that, a conjunctival incision of approximately 150° was performed in a conventional manner, followed by a sclerocorneal incision of approximately 150° along the corneal limbus in order to remove the nucleus employing the procedure of ECCE. After the scleral wound was sutured with 8‐0 silk and the cortex was aspirated with I/A, the purified sodium hyaluronate preparation (Heron eye viscoelastic agent 1%, Johnson & Johnson Surgical Vision, AMO Japan, Tokyo, Japan), which is a high molecular–weight viscoelastic substance, was applied to the anterior chamber for replacement and a 6‐mm diameter intraocular lens (IOL) was fixed in the capsular bag. The high molecular–weight viscoelastic substances were aspirated and Obisot (Alcon Japan Ltd., Tokyo, Japan) was administered into the anterior chamber, confirming that the iris miosis constricted to a perfect circle. The conjunctival wound was sutured with 8‐0 Vicryl (Ethicon Inc., Somerville, New Jersey, United States) and the surgery was completed with the conjunctival injection of antibiotics and Depo‐Medrol (Pfizer Inc., New York, New York, United States).

The conjunctival wound was normal 1 month following surgery. Corneal edema and inflammation in the anterior chamber were minimal and the IOL was nicely fixed in the capsule and centered well. Visual acuity was 0.02 (0.04 × S − 4.00D: C − 2.50DA × 135^°^) in her right eye due to the choroidal atrophy.

## 3. Intraoperative Observation Through NGENUITY

NGENUITY has normal filter images, yellow filter images, blue filter images, red filter images, and monochrome filter images. The digital screen of 55 in. was placed 1.2 m away from the primary surgeon, the assistant surgeon, and other doctors in the operation room. To emphasize the advantage of the digital visualization system, namely its ease of operation in dark places, we note that the operating room lights were turned off to optimize the visibility of the monitor. The setting values used are shown in Tables 1 and 2. Anterior capsule staining was performed using ICG due to insufficient transillumination with normal settings (Figure 2a). Although the contrast between the folded anterior capsule segment and the nucleus became clear when the CCC was created, in the CCC after staining the anterior capsule, it was difficult to distinguish between the folded anterior capsule segment and the anterior capsule (Figure 2b). Therefore, we adjusted the color contrast using NGENUITY. The anterior capsule was stained green by ICG, the nucleus was slightly yellowish‐brown due to a cataract with advanced nuclear hardness, and the yellow filter image was close to the color tone of the nucleus based on the color wheel (Figure 2c), whereas the blue filter image was close to the color tone of the anterior capsular segment that was folded when the CCC was created, with no enhancement of the color contrast and no visibility improvement (Figure 2d). The monochrome filtered image provided unclear color contrast between the anterior capsule segment and the anterior capsule folded at the time of CCC creation, and visibility was poor (Figure 3a). Therefore, in this case, as shown in Table 2, adding red emphasis to the monochrome mode enhanced the color contrast between the anterior capsule segment folded during CCC creation and the anterior capsule located behind it (Figure 3b), allowing the improved visibility to facilitate the completion of CCC (Figure 3c). With the use of NGENUITY, red reflexes were relatively reduced due to the characteristics of digital image processing and low‐light settings, contributing to the suppression of glare during surgery. Display conditions were adjusted as necessary to maintain sufficient visibility.

**Table 1:** NGENUITY Image Setting 1. “Normal filter image” mode, “blue filter image mode,” and “yellow filter image mode” settings in NGENUITY.

	Normal filter image	Blue filter image	Yellow filter image
Mode	1. Anterior	1. Anterior	1. Anterior
Camera	Standard	Standard	Standard
Light profile	Halogen	Halogen	Halogen
Gain	4	4	4
Channel	Na	Blue	Yellow
RGB	83,100,83	64,96,100	100,100,65
Brightness	48.00	47.80	47.80
Contrast	60.00	62.50	54.90
Gamma	1.30	1.30	1.20
Hue	2	8	2
Saturation	96	95	90‐

**Table 2:** NGENUITY Image Setting 2. “Monochrome filter image” mode and “monochrome filter image + red emphasis mode” settings in NGENUITY.

	Monochrome filter image	*M* *o* *n* *o* *c* *h* *r* *o* *m* *e* *f* *i* *l* *t* *e* *r* *i* *m* *a* *g* *e* + *r* *e* *d* *e* *m* *p* *h* *a* *s* *i* *s*
Mode	1. Anterior	1. Anterior
Camera	Standard	Standard
Light profile	Halogen	Halogen
Gain	4	4
Channel	Monochrome	Mono red
RGB	100,100,100	100,0,0
Brightness	47.80	47.80
Contrast	54.90	54.90
Gamma	1.20	1.30
Hue	2	2

Figure 2Surgery photos (a) At the start of surgery (before anterior capsule staining). (b–d) Anterior capsulotomy after anterior capsule staining ((b) normal filter image, (c) blue filter image, and (d) yellow filter image).

**Figure:**
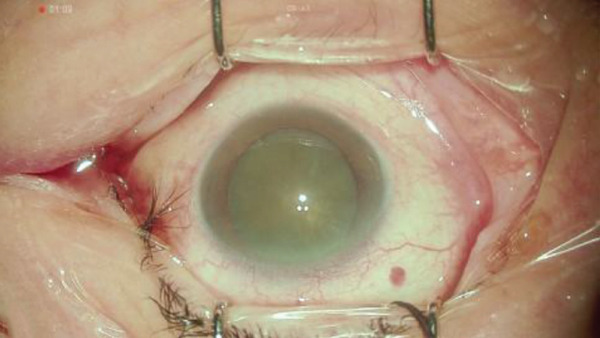
(a)

**Figure:**
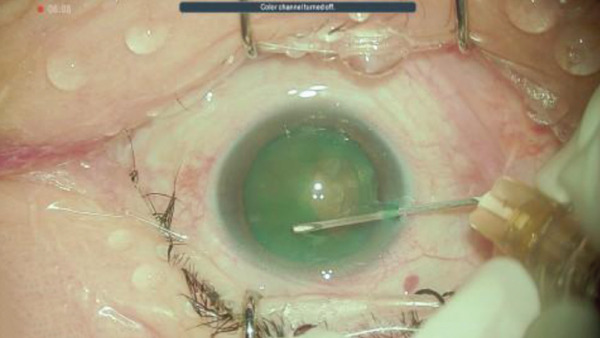
(b)

**Figure:**
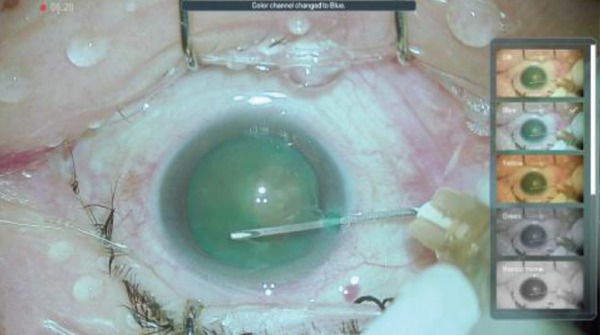
(c)

**Figure:**
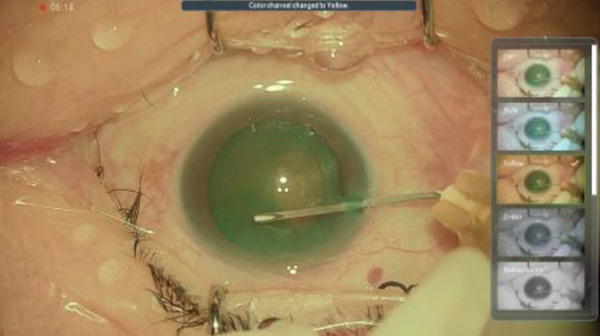
(d)

Figure 3Surgery photos (a, b) Anterior capsulotomy after anterior capsule staining. (a) Monochrome filter image. (b) Monochrome filter image + red emphasis. (c) Finish of anterior capsulotomy (monochrome filter image + red emphasis).

**Figure:**
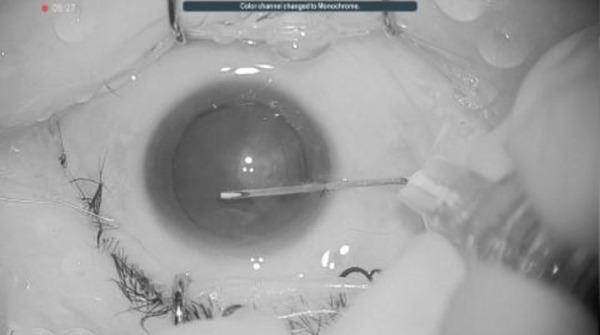
(a)

**Figure:**
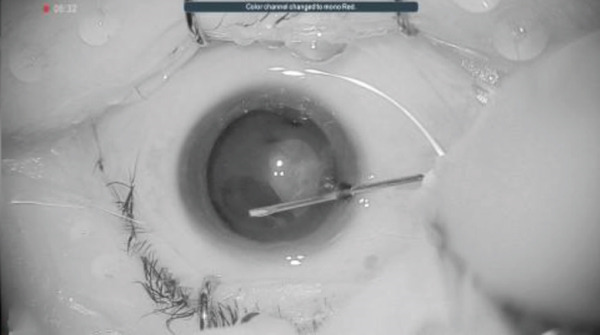
(b)

**Figure:**
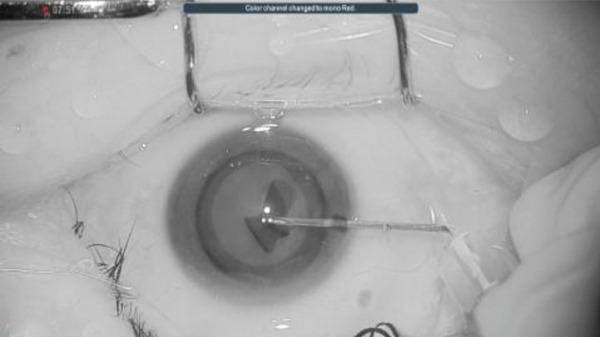
(c)

## 4. Discussion

The completion of CCC is critical to safe performance of cataract surgery; incomplete CCC increases the risk of complications such as decentration of an implanted IOL or tear formation in the posterior capsule [6]. Poor transillumination of the lens through an operating microscope might make it difficult to create a complete CCC in patients with cataracts with advanced nuclear sclerosis.

Among capsule‐staining reagents, for example, ICG, TB, and BBG, 0.5% ICG in BSS Plus is available in Japan under off‐label usage upon approval from the institutional review board [7]. The original report proposed 30‐s staining of the capsule with ICG [8], adjustment of color and contrast allowed us to shorten the staining duration to 5 s, which could reduce the risk of endothelial damage. There is no report available to discuss this point.

NGENUITY was launched in 2017 as the world′s first real‐time ophthalmic imaging system equipped with a high‐dynamic range (HDR) video camera. Images captured with a 3D video HDR camera are visualized as 3D images using a digital high‐resolution monitor and dedicated polarized glasses. The system features an HDR camera that captures a wide range of brightness levels and enables digital adjustment of intraoperative brightness, contrast, and color balance to optimize intraoperative visualization. Observation through NGENUITY enabled the digital adjustment of intraoperative brightness, contrast, gamma, and color tone. There are reports indicating that conventional microsurgeries and surgeries using NGENUITY have comparable safety and surgical results when it comes to cataract surgery [9–11]. There is no report on the effectiveness of adjusting color contrast with NGENUITY in order to improve visibility of the stained anterior capsule.

**Figure 4:**
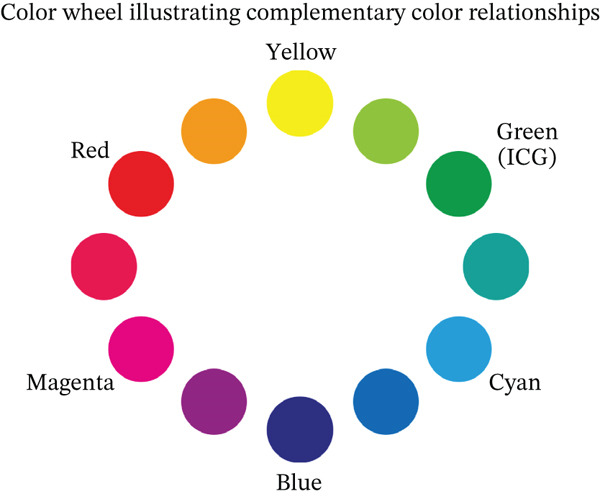
Color wheel illustrating complementary color relationships. Green and red are complementary (opposite on the hue circle).

In the present case, we adjusted the color contrast using NGENUITY. The anterior capsule was stained green by ICG, whereas the nucleus was yellowish‐brown due to cataract progression. Therefore, the contrast between the anterior capsule and the nucleus was enhanced in the yellow‐filter image, improving visibility (Figure 2d). Furthermore, adding red enhancement to the monochrome mode further improved the visibility of the ICG‐stained anterior capsule, facilitating the completion of CCC (Figure 3b,c). The background to this includes that, although the anterior capsule is stained green by ICG, the red reflex dominates the background under the surgical microscope, reducing the visibility of the anterior capsule due to color competition with green. Based on the color wheel, red is a complementary color to green, so the handling of the red component likely affects the perceived contrast of green (Figure 4). Although monochrome display reduces the influence of the red reflex and clarifies the shape and boundary of the anterior capsule, this alone did not provide sufficient color contrast in this case. In contrast, highlighting the red component as an independent channel separated the red from the background information, resulting in a relative increase in the contrast of the ICG‐stained anterior capsule (green), which contributed to the completion of CCC.

The safety and practical value of color contrast adjustments by using NGENUITY have been reported. For example, Park et al. demonstrated that customized color settings during digitally assisted macular surgery improved visualization of the internal limiting membrane without affecting postoperative visual outcomes [12]. Sandali et al. reported that monochrome digital filtering during cataract surgery enhanced image contrast without compromising safety [13]. Furthermore, Gualino et al. showed that digital HDR visualization using NGENUITY reduced glare and surgeon fatigue while maintaining surgical performance [14]. On the other hand, we have to further verify the effectiveness of the current method with surgeons and medical staff associated with visual abnormality. Furthermore, our limitations in the current report include that the surgery was conducted by a single surgeon, that there were no objective contrast indicators, that there were no control cases with the same difficulty and by the same surgeon, and finally that the impact of the learning curve was not considered.

In conclusion, adjusting the color contrast in HUS using NGENUITY for cataracts with advanced nuclear sclerosis is effective, taking into consideration complementary colors to further improve visibility when performing CCC with anterior capsule staining.

## Author Contributions

Y.T. and T.S. contributed to the design and conduct of the study. Y.T. contributed to the preparation of manuscript. N.I. contributed to the collection and management of data. S.S. contributed to the review of the manuscript.

## Funding

No funding was received for this manuscript.

## Disclosure

All authors agree to be accountable for aspects of the work. All authors attest that they meet the current ICMJE criteria for authorship.

## Ethics Statement

The patient provided written consent for publication.

## Conflicts of Interest

The authors declare no conflicts of interest.

## Data Availability

The data supporting the findings of this study are not publicly available due to patient privacy but are available from the corresponding author upon reasonable request.
